# Near Infrared Photoimmunotherapy; A Review of Targets for Cancer Therapy

**DOI:** 10.3390/cancers13112535

**Published:** 2021-05-21

**Authors:** Takuya Kato, Hiroaki Wakiyama, Aki Furusawa, Peter L. Choyke, Hisataka Kobayashi

**Affiliations:** Molecular Imaging Branch, Center for Cancer Research, National Cancer Institute, National Institutes of Health, Bethesda, MD 20892, USA; takuya.kato@nih.gov (T.K.); hiroaki.wakiyama@nih.gov (H.W.); aki.furusawa@nih.gov (A.F.); pchoyke@mail.nih.gov (P.L.C.)

**Keywords:** cancer, near-infrared photoimmunotherapy (NIR-PIT), target molecule, host immunity, cancer therapy

## Abstract

**Simple Summary:**

Near-infrared photoimmunotherapy (NIR-PIT) is a newly developed cancer treatment that uses an antibody-photoabsorber (IRDye700DX) conjugate (APC) that is activated by NIR light irradiation. A major benefit of NIR-PIT is that only APC-bound cancer cells that are exposed to NIR light are killed by NIR-PIT; thus, minimal damage occurs in adjacent normal cells. NIR-PIT has now been applied to many cancers expressing various cell-surface target proteins using monoclonal antibodies designed to bind to them. Moreover, NIR-PIT is not limited to tumor antigens but can also be used to kill specific host cells that create immune-permissive environments in which tumors grow. Moreover, multiple targets can be treated simultaneously with NIR-PIT using a cocktail of APCs. NIR-PIT has great potential to treat a wide variety of cancers by targeting appropriate tumor cells, immune cells, or both, and can be augmented by other immunotherapies.

**Abstract:**

Near-infrared photoimmunotherapy (NIR-PIT) is a newly developed cancer treatment that uses an antibody-photoabsorber (IRDye700DX) conjugate (APC) that is activated by NIR light irradiation. In September 2020, the first APC and laser system were conditionally approved for clinical use in Japan. A major benefit of NIR-PIT is that only APC-bound cancer cells that are exposed to NIR light are killed by NIR-PIT; thus, minimal damage occurs in adjacent normal cells. These early trials have demonstrated that in addition to direct cell killing, there is a significant therapeutic host immune response that greatly contributes to the success of the therapy. Although the first clinical use of NIR-PIT targeted epidermal growth factor receptor (EGFR), many other targets are suitable for NIR-PIT. NIR-PIT has now been applied to many cancers expressing various cell-surface target proteins using monoclonal antibodies designed to bind to them. Moreover, NIR-PIT is not limited to tumor antigens but can also be used to kill specific host cells that create immune-permissive environments in which tumors grow. Moreover, multiple targets can be treated simultaneously with NIR-PIT using a cocktail of APCs. NIR-PIT can be used in combination with other therapies, such as immune checkpoint inhibitors, to enhance the therapeutic effect. Thus, NIR-PIT has great potential to treat a wide variety of cancers by targeting appropriate tumor cells, immune cells, or both, and can be augmented by other immunotherapies.

## 1. Introduction

Near-infrared photoimmunotherapy (NIR-PIT) (Illiminox^TM^) is a newly developed cancer treatment that induces selective immunogenic cell death in targeted cells [1]. NIR-PIT utilizes an antibody–photoabsorber conjugate (APC) that is activated by NIR light, typically administered as laser light. A large body of research has developed around NIR-PIT, showing that it can kill many types of cancer cells by targeting unique transmembrane proteins overexpressed in cancer cells. Currently, a global phase III clinical trial of NIR-PIT for inoperable head and neck cancer patients is currently underway using an anti-epidermal growth factor receptor (EGFR)-antibody IRDye700DX (IR700) conjugate. In Japan, the first APC (ASP-1929, Akalux^TM^, Rakuten Medical Inc., San Diego, CA, USA) targeting EGFR and utilizing a near infrared laser system (BioBlade^TM^, Rakten Medical Inc.) was approved for clinical use in September 2020. Although NIR-PIT has been primarily developed to use antibodies against cancer membrane antigens, it can also kill normal host cells, especially those that promote cancer cell growth, by inhibiting the immune system locally [2].

Tumor-targeted NIR-PIT evokes a profound immune response. This can be augmented with various checkpoint inhibitors. The first such agent, ipilimumab, was a monoclonal antibody against cytotoxic T-lymphocyte-associated protein 4 (CTLA4) and was approved in March 2011 to treat patients with late-stage melanoma. This class of immune checkpoint inhibitor (ICI) has continued to expand over the last decade, with many ICIs being approved. Although these agents have been highly effective in some patients, immunotherapy-related side effects, termed immune-related adverse events (irAEs), have been widely reported in various organs. irAEs occur because ICIs activate immunity throughout the body, not just in the tumor. These side effects can cause syndromes that mimic autoimmune disease in normal organs. Ideally, a comprehensive cancer therapy would selectively kill tumor cells and activate local tumor immunity. None of the existing major therapies, surgery, radiation, or chemotherapy do this. Tumor- and immune cell-targeted NIR-PIT can kill tumor cells selectively and deplete local immune suppressor cells in the tumor beds. This leads to dramatically more active immune responses against tumors without inducing irAEs in non-target tissues.

Numerous NIR-PIT for cancer and immune cells have been attempted over the past decade, resulting in numerous research reports. This review summarizes these efforts and serves to compile this work into one review to provide an update on NIR-PIT against various effective molecular targets.

## 2. NIR-PIT

Historically, the term “photoimmunotherapy” meant targeted photodynamic therapy using antibodies conjugated with conventional photosensitizers such as hematoporphyrin based on cytotoxicity induced by reactive oxygen species (ROS) in the 1980s [3]. Various combinations of conventional photosensitizers and antibodies were tested to improve their delivery and selectivity. Due to the unfavorable chemical design that conjugated antibodies with hydrophobic chemicals, therapeutic effects were limited only on cells in vitro or tumors with intra-tumoral or intra-spatial administration in vivo. Therefore, such conjugates were not successfully applied to cancer therapy via systemic administration in vivo [4] because of insufficient delivery to the tumor due to rapid liver accumulation of conjugates promoted by the hydrophobicity of conventional photosensitizers, resulting in an insufficient accumulation of photosensitizers in the tumor [5]. Therefore, these photosensitizers have not been approved for clinical use. Furthermore, the cytotoxicity of such conjugates relied on ROS that mainly induced apoptotic cell death, yet caused non-specific damage on various parts of cells, including the cell membrane, in both targeted and adjacent non-targeted cells. In contrast, NIR-PIT (Illuminox^TM^) showed selective dedicated cytotoxicity on target cells based on a photo-induced ligand release reaction rather than ROS after the systemic intravenous injection of IR700-based APCs with various antibodies. Additionally, NIR-PIT is a hybrid therapy of direct cancer cell killing induced by NIR light exposure combined with rational immune activation. Therefore, NIR-PIT is totally different from the old “photoimmunotherapy” and is now defined as Illuminox^TM^.

Nanosized particles were used as carriers of photosensitizers due to the large loading capacity [6]. Nanosized drugs have an advantage on delivery into tumor beds through permeable tumor vessels and are then retained in the tumor bed. This process is known as the enhanced permeability and retention (EPR) effect [7]. However, the EPR effect offers less than a twofold increase in nanodrug delivery compared to critical normal organs with inhomogeneous intratumor micro-distribution, resulting in failure to cure most cancers [8]. Therefore, nanosized vehicles could be a good carrier for photothermal therapy (PTT), but would not rationally be a good carrier of photosensitizers for targeted photodynamic therapy (PDT). To overcome all these problems, NIR-PIT was developed as a novel cancer treatment with high cell selectivity with minimal toxicity to adjacent cells.

**Figure 1:**
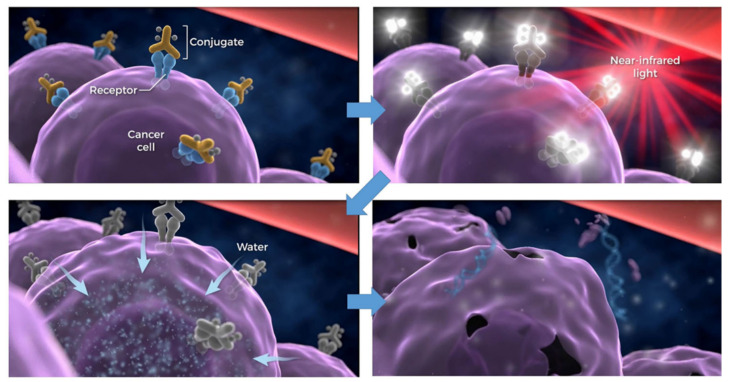
Scheme for cellular cytotoxicity induced by NIR-PIT. Antibody-photoabsorber conjugates bind to a specific cell membrane antigen. Immediately after NIR light exposure to APC, the water outside of the cells is flown into the target cell, leading to cell death. Adapted from Ref [10].

NIR-PIT utilizes an antibody conjugated with the NIR photon-absorbing silicon phthalocyanine dye IR700 [1,9,10]. When injected intravenously, APCs bind to a specific cell membrane antigen. Once bound, NIR light (approximately 690 nm) exposure kills only APC-bound target cells, sparing nearby cells which have no or minimal expression of the target antigen [1,9,11,12]. Immediately after NIR light exposure to APC, axial ligands of the IR700 molecule dissociate from the molecule, converting the APC from very hydrophilic to very hydrophobic. This change in the chemical characteristics of IR700 promotes conformational changes and aggregation of APCs that damage the cell membrane, causing weakening and eventually rupture of the cell (Figure 1). NIR-PIT can clearly be distinguished from conventional photodynamic therapy (PDT) or photothermal therapy (PTT), which rely on cytotoxic singlet oxygen and hyperthermia, respectively, and cause non-selective damage to light-exposed cells [13,14]. In addition to direct cancer-cell killing, NIR-PIT rapidly induces immunogenic cell death (ICD) [15,16], which initiates activation of the adaptive immune response, employing dead-cell-associated antigens, including calreticulin (CRT), adenosine triphosphate (ATP), high-mobility group box 1 (HMGB1), heat shock protein (Hsp) 70, and Hsp90 [17,18,19,20]. These danger signals activate local immature dendritic cells (DCs) to stimulate the presentation of tumor antigens, which are released from NIR-PIT-treated cancer cells. The DCs present these antigens to T cells, resulting in priming and educating naive T cells to become cancer-specific CD8^+^ T cells [15,21]. Therefore, NIR-PIT has the potential to reset anti-tumor host immunity due to ICD. These factors have contributed to the initial success of NIR-PIT.

## 3. NIR-PIT Targeting Cancer Cells

Initially, NIR-PIT was developed to target cells expressing EGFR, human epidermal growth factor receptor-2 (HER2), and prostate-specific membrane antigen (PSMA) [1]. Since those early days, NIR-PIT has expanded to target a variety of transmembrane proteins using monoclonal antibodies designed to bind to these antigens. Here, we review many of the targets that have been investigated for potential use in NIR-PIT using PubMed, with “photoimmunotherapy,” “near infrared,” and “IR700” as keywords (Table 1).

### 3.1. Epidermal Growth Factor Receptor

**Figure 2:**
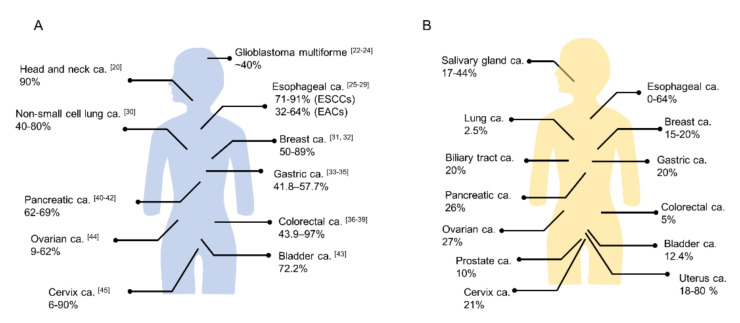
The positivity proportion of (**A**) EGFR and (**B**) HER2 expressions in various cancers (ca.; carcinoma, ESCC; esophageal squamous cell carcinoma, EAC; esophageal adenocarcinoma). ((**A**) The numbers in the brackets show reference number. (**B**) Quoted from references [50,51]).

EGFR, belonging to the erythroblastosis oncogene B (ErbB) family, is a transmembrane tyrosine kinase receptor [22]. Physiologically, EGFR regulates epithelial tissue development and homeostasis. EGFR mutation and/or overexpression is observed in several human cancers (Figure 2A) and EGFR-targeted therapy has become a routine part of the treatment of several cancers [23]. In several tumor types (e.g., head and neck, cervical, bladder, and esophageal cancer) EGFR overexpression has been associated with poor prognosis [24]. Hence, EGFR was considered a good target for early clinical trials of NIR-PIT. Multiple studies have confirmed the therapeutic efficiency of EGFR-targeted NIR-PIT both for in vitro testing and in mouse xenograft models [1,25,26,27,28,29]. Cetuximab, which is a chimeric IgG1 monoclonal antibody and a competitive inhibitor of EGFR ligand binding, was approved for head and neck squamous cell carcinoma (HNSCC), colorectal cancer, and non-small cell lung cancer (NSCLC) patients by the FDA. EGFR overexpression is very common in HNSCC patients (up to 90%) [30]. Based on the relative ease of applying light to HNSCC tumors and the poor outcomes for patients with recurrent disease with current therapies, this setting was the logical starting point for clinical translation of NIR-PIT. The first phase 1/2 clinical trial of NIR-PIT took place in patients with recurrent HNSCC using cetuximab-IR700 conjugates and successfully concluded in late 2017 [31]. The results suggested that EGFR-targeted NIR-PIT was more effective than currently recommended second- and third-line therapies for HNSCC. Cetuximab-IR700 was conditionally approved and registered for clinical use as the first EGFR-targeted NIR-PIT drug in Japan in 2020. Investigator-initiated clinical trials against EGFR-expressing esophageal and stomach cancers using cetuximab-IR700 started in 2019 for extending applications of the drug. Theoretically, EGFR-targeted NIR-PIT could be applied to other types of cancers with EGFR overexpression [32,33,34,35,36,37,38,39,40,41,42,43,44,45,46,47,48,49,50,51,52,53,54,55].

### 3.2. Human Epidermal Growth Factor Receptor 2

HER2, also known as HER2/neu, c-erbB2, or ERBB2, is a membrane tyrosine kinase and another member of the ErbB family [22]. HER2 was found to be amplified in a human breast cancer cell line 35 years ago [56]. HER2 does not bind EGF-like ligands, relying instead on heterodimerization with other ErbB family receptors for activation, which differs from other members of the ErbB family such as EGFR [57]. HER2 overexpression creates spontaneous receptor homodimers or heterodimers with other ErbB family receptors, resulting in activated oncogenic downstream signaling (e.g., PI3K/Akt/mTOR and MAPK) promoting cellular proliferation, survival, and angiogenesis [58]. In breast cancer patients, HER2 positivity rates have been reported in the range of 15% to 20% [59]. HER2 is an established therapeutic target of breast cancer and several drugs, including trastuzumab, pertuzumab, lapatinib, neratinib, and trastuzumab emtansine (T-DM1), have been approved for the treatment of HER2-positive breast cancer by the FDA. HER2 has also been detected in approximately 20% of gastric cancers [60,61]. Trastuzumab and fam-trastuzumab deruxtecan-nxki for HER2 positive gastric cancer patients have been approved by the FDA. Moreover, HER2 amplifications or mutations and HER2 overexpression have been reported in various solid tumor types (Figure 2B) [60]. For example, the overexpression rate of HER2 has been reported in 0% to 64% of esophageal cancer [62,63]. NIR-PIT utilizing trastuzumab-IR700 has been effective in xenograft models with a human breast cancer cell line, in pleural dissemination models by HER2 expressing NSCLC cells, in esophageal carcinoma cell lines in vitro, and in subcutaneous tumor models and disseminated peritoneal models with several ovarian cancer cell lines [1,64,65,66]. Combination therapy of HER2-targeted NIR-PIT and conventional chemotherapy such as 5-Fluorouracil (5-FU) rapidly induced significant tumor inhibition in a gastric cancer xenograft model [67]. Moreover, NIR-PIT with trastuzumab-IR700 and pertuzumab-IR700 conjugates showed stronger antitumor effects than with either mono conjugate in a gastric cancer xenograft model [68]. Therefore, HER2-targeted NIR-PIT is a promising therapy for various HER2 overexpressed tumors.

### 3.3. Cancer Stem Cell Markers

CD44, a non-kinase transmembrane glycoprotein, is a well-known marker of cancer stem cells (CSCs) and mediates intercellular adhesion, regulating epithelial to mesenchymal transition and cancer progression [69]. Various types of cancers, including most head and neck, gastric, lung, colorectal, and breast cancers, and hepatocellular carcinoma express CD44, making it a valuable diagnostic and prognostic marker [70]. Thus, CD44 is an important target for antibody-based therapies [71]. CD44-targeted NIR-PIT significantly suppressed tumor progression and prolonged survival in CD44-expressing syngeneic mouse models of oral squamous cell carcinoma [72]. Moreover, combined CD44-targeted NIR-PIT with programmed cell death protein 1 (PD-1) or CTLA4 checkpoint blockade was more effective in combination than as single therapies in syngeneic mouse models, including a minimally immunogenic tumor [21,73,74]. Combined CD44-targeted NIR-PIT with PD-1 blockade eradiated more than 70% of established tumors [21]. AC133, which is also a stem cell-specific glycosylation-dependent epitope of CD133, is a CSC marker found in many tumors [75]. AC133-targeted NIR-PIT suppressed tumor progression and prolonged survival for both subcutaneous and orthotopic tumor models of AC133 positive glioblastoma stem cells [76]. Thus, NIR-PIT targeting CSCs can induce significant therapeutic responses.

### 3.4. Prostate-Specific Membrane Antigen

PSMA is a well-established cell membrane marker of prostate cancer, and is expressed highest in poorly differentiated, metastatic, and hormone-refractory prostate cancer [77]. The expression level of PSMA is associated with the stage and grade of the prostate cancer, but the expression is low in normal tissues [78]. PSMA-targeted NIR-PIT significantly suppressed tumor growth and prolonged survival in mouse prostate tumor models [77]. Furthermore, PSMA-targeted small and bivalent antibody fragments, including diabodies (Db) and minibodies (Mb), were effective therapies using NIR-PIT. Localized prostate cancer would be amenable to focal therapy with a PSMA-directed NIR-PIT using light fibers inserted in the prostate. Because of its selectivity, it would be unlikely to damage nerves or sphincter muscles, yet could result in effective tumor control by direct killing and induction of anti-tumor host immunity. Therefore, NIR-PIT targeting PSMA is a reasonable approach for prostate cancer.

### 3.5. Carcinoembryonic Antigen

Carcinoembryonic antigen (CEA), a glycoprotein involved in cell adhesion, is already used as a tumor marker in various cancers, and the expression levels of CEA are related to prognosis in colorectal cancer patients [79,80,81]. More than 50 years after its initial discovery, CEA is still an excellent marker for differentiating colorectal cancer from normal tissues (98.8% of cases) and detecting positive lymph nodes [82]. Therefore, CEA is a promising target for cancer therapy. NIR-PIT targeting CEA suppressed tumor progression significantly not only in a xenograft model of gastric cancer but also in the orthotopic pancreatic tumor model without adverse effects [83,84]. For pancreatic cancer, complete tumor resection can improve overall survival. NIR-PIT could be used as an adjuvant to surgery, treating any residual pancreatic cancer. This was tested in a patient-derived orthotopic xenograft model using CEA-targeted NIR-PIT [85]. Moreover, surgery with CEA-targeted NIR-PIT reduced recurrence by targeting residual cancer cells [86]. Thus, CEA-targeted NIR-PIT could be an adjuvant to conventional cancer therapies.

### 3.6. Podoplanin

Malignant pleural mesothelioma (MPM) is a malignant tumor that originates from mesothelial cells and has an extremely poor prognosis [87,88]. Podoplanin (PDPN) is a type I transmembrane glycoprotein that is expressed in lymphatic endothelial cells, type I alveolar epithelial cells, and podocytes of the glomeruli. The antibody (D2-40) for PDPN has been used for a long time as a specific pathological diagnostic marker to distinguish lymphatic vessels from blood vessels and to verify the presence of MPM [89,90,91]. PDPN is also upregulated in various tumors, including MPM, angiosarcomas, chondrosarcomas, osteosarcomas, germ-cell tumors, gliomas, glioblastomas, dysgerminomas of the ovary, and squamous cell carcinomas of the skin, esophagus, lung, cervix, and head and neck [92,93]. For NIR-PIT, the lung and thoracic cavity can effectively transmit NIR light over long distances, because it consists mainly of air. PDPN-targeted NIR-PIT using the NZ-1 antibody demonstrated tumor suppression in both xenograft and orthotopic MPM models [87].

### 3.7. Mesothelin

Mesothelin (MSLN) is a cell-surface glycoprotein and tumor differentiation marker expressed in several tumors, including lung cancer, pancreatic cancer, ovarian cancer, and MPM [94]. MSLN can be used as a systemic diagnostic biomarker and is a target for antibody-based therapies [94,95]. MSLN-targeted NIR-PIT has been shown to be effective in mouse xenograft models [96]. NIR-PIT targeting MSLM is a promising candidate for the treatment of mesothelin-expressing tumors.

### 3.8. Glycoprotein A33 Antigen

Glycoprotein A33 antigen (GPA33) could be another target of NIR-PIT in colorectal cancer because it is highly expressed in over 95% of human colorectal cancers, more than 60% of gastric cancers, and 50% of pancreatic cancers [97]. Additionally, GPA33 exhibits limited expression in the normal intestinal epithelium [98]. GPA33-targeted NIR-PIT demonstrated tumor growth suppression in a xenograft model [99].

### 3.9. Tumor-Associated Calcium Signal Transducer 2

Tumor-associated calcium signal transducer 2 (TROP2) is a 46kD glycoprotein that plays a multifunctional cellular role, including transducing of cytoplasmic calcium. TROP2 is overexpressed in many epithelial cancers, including gastrointestinal tumors, cholangiocarcinoma, and prostate and pancreatic cancer [100]. Furthermore, it corelates with a poor prognosis in various cancers [101]. TROP2-targeted NIR-PIT inhibited tumor growth in cholangiocarcinoma and pancreatic cancer models [102]. Thus, TROP2-targeted NIR-PIT is an attractive candidate for TROP2 expressing tumors.

### 3.10. Cadherin-17

Cadherin-17 (CDH-17), a cell surface biomarker specific for gastrointestinal cancers, is a cell−cell adherent junctional molecule and plays an important role in cancer cell adhesion, progression, and metastasis [103]. Moreover, CDH-17 is highly expressed on colorectal, gastric, and pancreatic adenocarcinoma [104]. CDH17-targeted NIR-PIT inhibited tumor growth in a pancreatic and gastric xenograft model [105]. Thus, CDH-17 is another promising agent for NIR-PIT in gastrointestinal cancers.

### 3.11. Delta-Like Protein 3

Delta-like protein 3 (DLL3) is a potential therapeutic target molecule for small cell lung cancer (SCLC) and other neuroendocrine tumors that is minimally expressed in normal tissues [106]. However, rovalpituzumab tesirine, which is the first antibody targeting DLL3, was terminated on August 2019 because of the failure of the TAHOE (NCT03061812) and MERU (NCT03033511) clinical trials [107]. Notwithstanding this result, NIR-PIT targeting DLL3 in SCLC xenograft model showed marked antitumor effects [108]. NIR-PIT targeting DLL3 using rovalpituzumab could easily be translatable into the clinic since rovalpituzumab has already been used in a human clinical trial [109].

### 3.12. Glypican-3

Glypican-3 (GPC3), a 65-kDa membrane-bound heparin sulfate proteoglycan that is highly expressed in hepatic cell carcinoma (HCC) but not in normal tissue, is being evaluated as a potential therapeutic target for HCC [110,111]. GPC3-targeted NIR-PIT inhibited tumor growth compared to an untreated control in a xenograft model [112]. Moreover, the combination of GPC3-targeted NIR-PIT and the nanoparticle albumin-bound paclitaxel enhanced the therapeutic effect [113].

### 3.13. c-KIT

Gastrointestinal stromal tumors (GISTs) are the most common submucosal tumors (SMTs) of the alimentary tract [114]. SMTs are derived from mesenchymal cells and are generally covered with normal mucosa, so it is difficult to distinguish GIST from other types of SMTs by conventional endoscopy [115,116]. However, c-KIT is expressed in over 90% of GISTs on the cell membrane; therefore, it can be a molecular target for NIR-PIT [115,116]. Moreover, c-KIT-targeted NIR-PIT using anti-CD117 antibody induced acute necrotic cell death and was very effective in a GIST tumor model [115]. Thus, NIR-PIT targeting c-KIT could be a novel effective technology for GISTs.

### 3.14. CD20

Lymphoma is a diverse group of B-cell, T-cell, and natural-killer cell tumors, but the majority of lymphomas (approximately 90%) are of B-cell origin [117]. Malignant cells that originate from the B-cell lineage often express B-cell differentiation antigens such as CD19 and CD20, which can be targeted by monoclonal antibodies [118]. Utilizing anti-CD20 monoclonal antibody rituximab, CD20-targeted NIR-PIT against B-cell lymphoma significantly inhibited tumor growth [119], and the therapeutic effect of NIR-PIT was better than that of radioimmunotherapy against a xenograft model of aggressive B-cell lymphoma [120].

### 3.15. Cutaneous Lymphocyte Antigen and CD25

Mycosis fungoides is a rare cancer, however, it is the most common subtype of cutaneous T-cell lymphoma [121]. It is reported that mycosis fungoides cells express cutaneous lymphocyte antigen (CLA) [122]. CLA-targeted NIR-PIT specifically killed a mycosis fungoides cell line in vitro [123]. Thus, NIR-PIT could treat such locoregional lymphomas. Additionally, CD25, which is an α-subunit of interleukin-2 receptor and overexpressed in most cases of adult T-cell leukemia/lymphoma (ATLL), is thought to arise from the regulatory T cell (Treg) [124]. Therefore, CD25 is potentially a good target molecule for cutaneous lesions of ATLL and for Treg killing.

### 3.16. CD146

CD146 has been identified as a melanoma cell adhesion molecule. The expression of CD146 in melanoma is high and is found in 70% of primary melanomas and 90% of their lymph node metastases [125]. Wei et al. reported that CD146-targeted NIR-PIT inhibited tumor growth in a CD146-positive melanoma xenograft model [126].

### 3.17. H-Type Lectin/β-D-Galactose Receptors

Non-antibody mediated NIR-PIT has proven to be more difficult than antibody mediated NIR-PIT. However, there are some advantages of using non-antibody targeting agents such as galactosyl serum albumin (GSA), because GSA as a target is ubiquitous among ovarian cancers, whereas it is difficult to find a single antibody that will bind to all types of ovarian cancers. GSA is comprised of galactose molecules conjugated via carboxyl groups to an albumin molecule, and it binds to β-D-galactose receptors, also called H-type lectins, which are overexpressed on the surface of many ovarian tumors [127]. Although a large amount of NIR light exposure was needed for effective NIR-PIT therapy because GSA ligands were quickly internalized after binding to β-D-galactose receptors, GSA-targeted NIR-PIT specifically killed ovarian cancer cells (SHIN3) in vitro and suppressed tumor growth in a peritoneal disseminated model [128]. This may be especially useful when specific antibodies are not available for ovarian cancers. The development of similar non-antibody-based forms of NIR-PIT could therefore be an effective way of extending the utility of this novel therapy to other tumors.

### 3.18. Programmed Death-Ligand 1

PD-1 (CD279) is a T-cell immune checkpoint that suppresses autoimmunity, leading to immune tolerance of cells expressing programmed death-ligand 1 (PD-L1, CD274) [129]. Upregulated PD-L1 on these cells contributes to the development of T-cell exhaustion [130]. PD-L1 is expressed in various types of normal cells (including placenta, vascular endothelium, pancreatic islet cells, muscle, hepatocytes, epithelium, and mesenchymal stem cells, as well as on B cells, T cells, DCs, macrophages, and mast cells), but it is overexpressed in many cancer cells, including melanoma, renal cell carcinoma, NSCLC, thymoma, and ovarian and colorectal cancer [129,131]. Furthermore, PD-L1 overexpression is associated with a poor prognosis [132]. Several antibodies against PD-1/PD-L1 have recently been developed for clinical application by the FDA. PD-L1-targeted NIR-PIT using avelumab, which is a fully humanized IgG1 anti-PD-L1 monoclonal antibody (mAb), significantly inhibited tumor growth and prolonged survival in a xenograft model [133]. Thus, PD-L1-targeted NIR-PIT is another potential cancer therapy.

## 4. NIR-PIT Targeting Non-Cancer Cells

Cancer immunotherapy is aimed at activating or boosting the activation of the immune system to attack cancer cells through endogenous immunity mechanisms [134]. The pioneering checkpoint inhibitor ipilimumab, which is a mAb that targets CTLA4, was approved for advanced melanoma in 2011 [135]. Over the past several years, other novel immunotherapies, including checkpoint inhibitor mAbs that target PD-1 or PD-L1 and chimeric antigen receptor T-cell therapies, have been developed and approved for clinical use [134,136,137,138]. Despite these major advances, the clinical use of immunotherapies faces several challenges related to both efficacy and safety. Although the immune upregulation provoked by these therapies results in an enhanced antitumoral response, it can also cause numerous inflammatory and autoimmune manifestations that can be severe, and on occasion, fatal [139]. For that reason, an alternative approach is to inhibit or kill immunosuppressive cells only within the tumor bed but not systemically. Hence, a new type of NIR-PIT targeting non-cancer immune-related cells is being developed.

### 4.1. CD25

Within the tumor tissue, T cells and natural killer (NK) cells that can recognize cancer cells are often present in large numbers, but their cytotoxic function is suppressed by nearby immunosuppressor cells such as Tregs [140,141]. Controlling tumor-infiltrating CD4^+^CD25^+^Foxp3^+^ Tregs has been considered an essential step for enhancing anticancer immune reactions [142,143]. However, when targeting CD25, activated effector cells that also express CD25 are depleted in addition to Treg cells, and this could interfere with activation of effector cells [144]. Furthermore, systemic blockade of immunosuppressive regulatory mechanisms runs the risk of inducing autoimmune adverse events. Thus, CD25-targeted NIR-PIT was developed to selectively deplete CD4^+^CD25^+^Foxp3^+^ Tregs in tumor beds to induce the activation of antitumor effector cells [2]. CD25-targeted NIR-PIT caused necrotic cell death against CD25-expressing HT-2-A5E cells in vitro, but in other cancer cells, no therapeutic effect was detected. In an in vivo syngeneic model, CD25-targeted NIR-PIT induced regression of treated tumors with rapid activation of tumor-infiltrating CD8^+^ T and NK cells and activation of antigen-presenting cells. Furthermore, the therapeutic effects of CD25-targeted NIR-PIT extended to distant non-treated tumors in a tumor-specific manner, because activated CD8^+^ T and NK cells were increased in the non-treated tumor after this therapy.

CD25 is also known as the IL-2 receptor α-chain. The IL-2 receptor is expressed not only on Tregs but also on activated lymphocytes such as CD8^+^ T cells and NK cells [145]. Remaining circulating anti-CD25-mAb-IR700 could block these receptors, hindering T-cell expansion [146]. Okada et al. reported that anti-CD25-F(ab’)_2_ NIR-PIT was more effective than anti-CD25-IgG NIR-PIT in suppressing tumor growth and inducing abscopal effects in vivo. The faster clearance of anti-CD25-F(ab’)_2_-IR700 and the absence of antibody-dependent cellular cytotoxicity (ADCC) or complement-dependent cellular cytotoxicity (CDC) contributed to the therapeutic efficacy of CD25-targeted NIR-PIT by minimizing the circulating APCs with their ability to block the IL-2 receptor. Thus, local CD25-targeted NIR-PIT produces tumor type-specific, systemic antitumor effects, which alter the growth of distant tumors of the same tumor type. Since expression and regulation of CD25 has been well investigated and known, local CD25-targeted NIR-PIT is a promising method for reducing immunosuppression caused by Tregs and thus augmenting effector cell-mediated cancer cell killing.

### 4.2. CTLA4

A monoclonal antibody against CTLA4 was approved as an immune checkpoint inhibitor for clinical use in 2011. CTLA4 and CD28 are expressed on T cells and share the binding ligands CD80 and CD86 [147,148]. CD28 is constitutively expressed on T cells, but CTLA4 is expressed only on activated T cells and binds to CD80/86 on antigen-presenting cells with higher affinity than CD28 [148,149,150]. Thus, blockade with an anti-CTLA4 antibody of the binding between CTLA4 and CD80/86 promotes priming in lymph nodes and enhances antitumor immunity [151]. CTLA4 antibody has demonstrated dramatic responses in some patients, but it has also been reported that irAEs were observed in up to 90% of patients treated with a CTLA4 antibody [132,152]. To address this problem, CTLA4-targeted NIR-PIT was developed to deplete CTLA4-expressing cells only in the tumor microenvironment. CTLA4-targeted NIR-PIT caused a T-cell mediated antitumor effect in four syngeneic tumor models of cancer and eradiated more than 50% of established tumors. The balance of effector T cells and Tregs after CTLA4-targeted NIR-PIT was improved, favoring effector intratumoral CD8^+^ T cells and resulting in T-cell activation in regional lymph nodes. Furthermore, CTLA4-targeted NIR-PIT induced an abscopal effect in over half of the tumor-bearing mice. It is believed that residual anti-CTLA4-IR700 might enhance antitumor immunity by promoting T-cell priming. Thus, CTLA4-targeted NIR-PIT is a promising antitumor therapy. However, CTLA4 exists in the cytoplasm in both normal and cancer cells to a much larger extent than CTLA4 expressed on the cellular membrane. Therefore, it is still unclear how the effects of CTLA4-targeted NIR-PIT affect these cells with the large reserve of intracellular CTLA4.

### 4.3. CD206

Tumor-associated macrophages (TAMs) play essential roles in tumor invasion and metastasis, and contribute to drug resistance [153]. TAM levels are correlated with poor prognosis in various human cancers and are a major factor in the development of resistance to chemo- or radiotherapy, as well as tumor recurrence [154]. TAM-targeted NIR-PIT using an anti-CD206 antibody inhibited the growth of subcutaneous tumors and prevented their metastasis to the lungs in a tumor model in which M2 macrophages were induced within tumors by sorafenib treatment [153].

### 4.4. Fibroblast Activation Protein

Cancer-associated fibroblasts (CAFs) are the most abundant cell population in the tumor microenvironment (TME), and are thought to play an essential role in tumor progression, angiogenesis, metastasis, and immunosuppression [155,156,157,158]. Fibroblast activation protein (FAP), a specific surface marker of CAFs, is recognized as the leading target molecule for CAFs. Several reports have already demonstrated the efficacy of systemically depleting or targeting FAP^+^ cells in reducing tumor and stroma growth; however, serious side effects have been observed in these approaches, such as cachexia and anemia [159]. Since rapid cell death was induced specifically and locally for CAFs, FAP-targeted NIR-PIT suppressed tumor progression and overcame chemoresistance in an esophageal tumor model without adverse effects [160]. Thus, the combined use of FAP-targeted NIR-PIT with conventional anticancer treatments can be expected to provide a more effective treatment strategy [157,160].

### 4.5. CD47

CD47, a cell-surface protein that prevents cancer cell phagocytosis via the “don’t eat me” signaling, is highly expressed on several human cancers, including acute myeloid leukemia, non-Hodgkin’s lymphoma, and bladder cancer [161]. CD47-targeted NIR-PIT increased direct cancer cell death and phagocytosis, resulting in inhibited tumor growth and improved survival in a murine xenograft model of human bladder cancer [162]. Similarly, calreticulin is a cell surface protein that confers an “eat me” signal known to counterbalance the “don’t eat me” signal conferred by CD47 [161]. For this reason, increased prophagocytic calreticulin expression through CD47-targeted NIR-PIT could provide additional tumor cytotoxicity.

### 4.6. Vascular Endothelial Growth Factor Receptor

Angiogenesis is regulated principally by interactions between vascular endothelial growth factors (VEGFs) and vascular endothelial growth factor receptors (VEGFRs), and plays a key role in cancer growth and metastasis in various malignant tumors [163,164]. The overall survival in patients with metastatic or unresectable gastric cancer was significantly improved by treatment with an anti-VEGFR-2 antibody, ramucirumab, which was approved by the FDA [165,166]. Applying the theory of NIR-PIT, VEGFR2-targeted NIR-PIT led to reduced tumor growth and vascularity without phototoxic effects [167,168]. The application of NIR-PIT utilizing ramucirumab is anticipated to lead to an enhanced antitumor effect.

## 5. NIR-PIT Targeting Forced Expression of New Specific Transmembrane Antigens

When no good target antigen exists on a cancer cell, gene transfer technology could be used to make cells express an artificial target antigen, which could then be easily targeted with an APC. For example, HER2-targeted NIR-PIT has promising antitumor effects in HER2-positive cancers [1]. However, HER2 positivity rates in breast cancer patients have been reported in the range of 15% to 20% [59]. The HER2-positive proportion in gastric cancers was estimated at only 12.2 to 22.1% [169], and gastric cancer itself exhibits intertumoral HER2 heterogeneity [170]. To overcome the lack of molecular target antigens, a replication-deficient adenoviral vector containing a gene that encodes the HER2 extracellular domain (HER2-ECD) was developed, and resulted in expression with HER2-ECD on the surface of HER2-negative breast and gastric cancer cells [171]. The expressed HER2-ECD did not trigger HER2 signaling pathways because of the lack of the HER2 intracellular kinase domain. Although exogenous HER2-ECD expression had no obvious effect on the signaling pathway, ADCC activity by trastuzumab was strongly enhanced. These results indicate that artificial exogenous domains expressed on the cell surface can be targets for NIR-PIT cancer treatment. Using this theory, HER2-targeted NIR-PIT induced direct cell membrane destruction of HER2-ECD-transduced HER2-negative breast cancer cells [172]. Furthermore, in a mouse model of peritoneal dissemination of HER2-negative gastric cancer, the combination of HER2-ECD administration and HER2-targeted NIR-PIT inhibited peritoneal metastasis and prolonged survival without severe adverse effects [173]. However, an obvious flaw with this strategy is that it might be difficult to transfer a gene to all cancer cells. Therefore, none of the athymic mice were cured with either HER2-ECD-targeted ADCC or NIR-PIT. However, under normal immunity, as a consequence of the massive ICD induced by NIR-PIT, patients might acquire immunity against neo-antigens on unresponsive cancers without expressing recognizable transmembrane antigens other than HER2, and this could result in successful treatment of the cancers. Thus, the combination of gene transfer technology in which a target antigen is introduced into the cell followed by targeted NIR-PIT would expand and potentiate molecular-targeted therapy even for target-negative or attenuated cancer cells.

## 6. Combination Therapy of NIR-PIT

NIR-PIT can simultaneously target two or more kinds of cells by co-injecting two or more different APCs, followed by exposure to NIR light. The combination approach for NIR-PIT is greatly advantageous, as it allows for targeting a broader spectrum of tumors more effectively, irrespective of enrichment of any one specific target. The combination NIR-PIT using panitumumab-IR700 and trastuzumab-IR700 was more efficacious than either monotherapy alone in a bladder cancer xenograft model [28]. Furthermore, even if the target is similar, when two different epitopes of HER2 were targeted on human gastric cancer cells using trastuzumab-IR700 (against domain IV of HER2) and pertuzumab-IR700 (against domain II of HER2), the combination NIR-PIT demonstrated synergy in a HER2-expressing xenograft model [68]. Moreover, this theory of NIR-PIT can be applied to target not only other tumor markers but also other types of cells in combination. Combined CD44- and CD25-targeted NIR-PIT simultaneously eliminates cancaer cells and Tregs in tumors. The combined NIR-PIT induced significant tumor growth inhibition and resulted in prolonged survival compared to either type of NIR-PIT alone in several allograft models [174]. Thus, this combined regimen of NIR-PIT is another effective approach, especially for tumors with high target antigen expression and with abundant Tregs, to induce long-term antitumor immunity.

## 7. Conclusions

**Figure 3:**
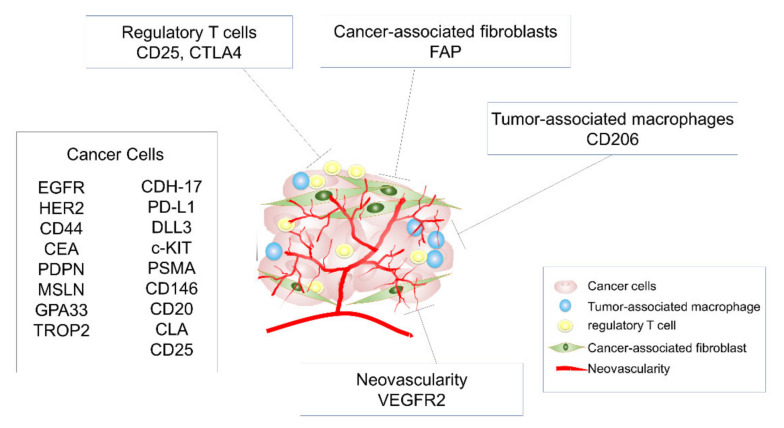
Target molecule of NIR-PIT developed for cancer therapy. NIR-PIT can target not only various surface molecules expressing cancer cells but also immunosuppressive cells that compose the tumor microenvironment, such as regulatory T cells, tumor-associated macrophages, cancer-associated fibroblasts, and neovascularity.

We reviewed various antibodies that have been used for NIR-PIT, emphasizing the broad spectrum of tumor antigens that can be targeted and introducing targeting of cells in the tumor microenvironment (Figure 3). NIR-PIT has the advantage of adapting to cancers in any organs where NIR light reaches. In the near future, NIR-PIT has great potential to treat a wide variety of cancers by targeting appropriate cancer cells, immune cells, or both.

## Figures and Tables

**Table 1:** Correspondence of molecular targets for each tumor in NIR-PIT targeting cancer cells.

Malignant Neoplasma	Target Moleclue										
	EGFR	HER2	CD44	CEA	PDPN	MSLN	GPA33	TROP2	CDH-17	PD-L1	Cancer Specific Target
Glioblastoma multiforme	+				+					+	
Head and neck Ca.	++	+	++		+					++	
Lung Ca.	++	±	+	+	+	+				++	DLL3 (SCLC)
MPM					+	+					
Breast Ca.	++	+	+	+						+	
Gastrointestinal Ca.											
Esophageal Ca.	++		+	±	+					+	
Gastric Ca.	+	+	++	+			+	+	+	+	
Colorectal Ca.	+	±	+	++			+	+	+	+	
Hepatic cell Ca.			+							+	GPC-3
cholangiocarcinoma			+	+				+		+	
Pancreatic Ca.	+	+	+	+		+	+	+	+	+	
GIST											c-KIT
Bladder Ca.	++	+	+							+	
Prostate Ca.		±	+							+	PSMA
Cervical Ca.	++	+	+	±	+					+	
Ovarian Ca.	+	+	+	+	+	+				+	
Malignant melanoma										++	CD146
Lymphoma										++	CD20, CD25, CLA

Ca., cancer; EGFR, epidernal growth factor receptor; HER2, human epidermal growth factor receptor-2; CEA, carcinoembryonic antigen; PDPN, podoplanin; MSLN, mesothelin; GPA33, glycoprotein A33 antigen; TROP-2, tumor-associated calcium signal transducer 2; CDH-17, cadherin-17; PD-L1, programmed death-ligand 1; DLL3, delta-like protein 3; GPC-3, glypican-3; PSMA, prostate-specific membrane antigen; CLA, cutaneous lymphocyte antigen; MPM, malignant plueral mesothelioma; GIST, Gastrointestinal stromal tumors; ++, highly expressing; +, moderately expressing; ±, weakly expressing.

## Data Availability

No new data were created or analyzed in this study. Data sharing is not applicable to this article.
